# Impact of maternal melatonin suppression on forced swim and tail suspension behavioral despair tests in adult offspring

**Published:** 2015

**Authors:** SE Voiculescu, AE Rosca, V Zeca, L Zagrean, AM Zagrean

**Affiliations:** * Division of Physiology & Fundamental Neuroscience, “Carol Davila” University of Medicine and Pharmacy, Bucharest, Romania

**Keywords:** melatonin, neurodevelopment, continuous light, depression

## Abstract

Melatonin is an essential hormone, which regulates circadian rhythms and has antioxidative and anticarcinogenic effects. As melatonin secretion is suppressed by light, this effect was examined on the offspring of the Wistar rat females exposed to continuous light (500 lux) during the second half of the pregnancy (day 12 to 21). Control rats were kept under a 12:12 light-dark cycle. The resulted male offspring have been behaviorally assessed for depression after postnatal day 60 by using Forced Swim Test (FST) and Tail Suspension Test (TST). Animals resulted from the melatonin deprived pregnancies have developed an abnormal response in the TST, but a normal FST behavior. Also, TST active movement was different in the melatonin suppression group compared to the control group. These findings suggest that intrauterine melatonin deprivation might be linked to the depressive like behavior in adult male offspring.

## Introduction

Melatonin is an indoleamine secreted in a circadian manner by the pineal gland and has key actions in human physiology. The role of melatonin in embryo-fetal development is not known, but highly suggested by the expression of melatonin receptors in the embryo and fetus. Their presence has been shown in the early development, with a high expression rate in the nervous system [**1**-**3**]. In the past years, a new emerging theory has been incriminating melatonin as being the mediator of the foetal programming phenomenon [**4**].

Disturbances in melatonin secretion, the signaling molecule of environmental darkness, have been involved in various psychopathologies in humans (seasonal affective disorder, bipolar disorder, depression, bulimia, anorexia, schizophrenia, panic disorder, obsessive compulsive disorder), but it is not clear yet if melatonin deficiency has an involvement in the etiopathology of these multifactorial disorders [**5**].

Recently, it was shown that melatonin MT1 receptor knockout mice have decreased mobility in the forced swim test (FST) and tail suspension test (TST), sustaining the presence of behavioral and neurobiological changes corresponding to the human melancholic depression [**6**].

The role of melatonin in depression is sustained by the fact that agomelatine, a melatonergic antidepressant with both MT1/ MT2 melatonin receptor agonist properties and selective antagonism to 5-HT2c serotonin receptor, showed its efficacy in many clinical studies of depressive disorders, and mostly in major depressive disorder, through the significant reduction in depression clinical scores (HAM-D) [**7**-**10**].

At present, the experimental evidence linking prenatal melatonin signaling to adult physiology and behavior is poor [**11**]. We hypothesized that melatonin cycle disruption *in utero* would induce neurodevelopmental changes which may lead to depressive behavior in the male offspring. 

## Materials and methods

Experiments were performed using male and female Wistar albino rats. All animal procedures were carried out with the approval of the local ethics committee for animal research of Carol Davila University of Medicine and Pharmacy, Bucharest, Romania, in accordance with the European Communities Council Directive 86/609/EEC on the protection of animals used for scientific purposes.

Melatonin suppression in dams was achieved by functional pinealectomy model [**12**-**14**], obtained by continuous light exposure of pregnant Wistar rats between embryonic days 12-21 (E12-E21). Control rats were kept under a normal light/ dark cycle (06:00-18:00 hours). All the animals were kept under controlled light (500 lux), with free access to rat chow and water. 

After birth, the male pups (N=8 for control group and N=8 for experimental melatonin deprived group) were raised under normal circadian conditions, with a 12:12 light/ dark cycle. After PN60 (postnatal day 60), they were tested for depressive-like behavior, using two classic tests: TST and FST, based on the same principle of measurement of the immobility duration when rodents are exposed to an inescapable situation. Behavioral testing was performed in the evening, at the beginning of the dark period [**15**,**30**]. The rats were videotaped and analysis was manually done by two observers. 

***Forced swim test protocol***

The test was conducted according to modified Porsolt et al. protocol (1977) in order to be used for rats [**16**,**31**].

The FST is a 2-days procedure in which rats swim under conditions in which escape is not possible. On the first day, the rats were placed in a 35 cm tall, 30 cm diameter cylinder, filled up to 21.5 cm with water at 24±0.5°C. After 15 min, the rats were removed from water, dried with towels and placed in a warmed enclosure. The 5-min test sessions were succeeded at 24 h and videotaped from above the cylinders. The frequency of mobility and immobility of the animals was analyzed, as well as the presence of differences in the active moves that they have performed during the test. Two observers did assessment, by manually recording the rat’s behavior once at 5 seconds during the 5 minutes test trial. 

The three behaviors that have been recorded are (1) immobility: the lack of motion of the whole body, except for small movements necessary to keep the animal’s head above the water; (2) climbing: vigorous movements with the forepaws in and out the water, usually directed against the wall of the cylinder; (3) swimming: when the animal is swimming without touching the walls of the recipient. Diving was not taken into account within the analysis.

***Tail suspension test protocol***

This tests’ protocol is conceptually related to the forced swimming test, except that immobility is induced by suspending the mice by the tail. After initially trying to escape by vigorous movements, at some point, rats will become immobile. The duration of immobility is reduced by antidepressant medication and it is usually used to test the efficacy of different drugs. This procedure has several advantages over the forced swim procedure: no hypothermia is induced; no special post-experimental treatment (rubbing down, a warmed environment) is required [**17**,**32**].

Rats were individually suspended from the tip of the tail. Duration of mobility and immobility were determined, as well as variations of the active moves of the rats during testing (presence or absence of certain types of movements). Each rat was tested in a single trial that has lasted for 6 minutes.

**Statistical analysis**

Continuous data was expressed as mean ± SD. The homogeneity of variance for all parameters was analyzed by Kolmogorov-Smirnov test. As all variables had a Gaussian distribution, “T-test” was performed for comparison between groups. SPSS (Statistical Package for Social Sciences, Inc., Chicago, Illinois) Windows 20.0 software was used for a statistical analysis. A two-sided p-value < 0.05 was considered statistically significant.

## Results

Mean values of parameters describing the performed behavioral tests (FST and TST) are illustrated in **Table 1**.

**Table 1:** Mean values of different behavioral parameters in the two groups of rats. FST - Forced Swim Test; TST - Tail Suspension Test; IMOB - frequency of immobility; SWIM - frequency of swimming; CLIMB - frequency of climbing; ACTIVE MOVES - the mean time during any type of active moves; IMMOBILITY - the mean duration of immobility; SPINNING - the mean time during spinning behavior; C group - control group; MD group - melatonin deprived

FST	IMOB	SWIM	CLIMB
C group	19.38 ± 7.8	4.00 ± 2.73	36.88 ±7.99
MD group	10.25 ± 9.8	4.75 ± 6.13	45.00 ± 13.8
TST	ACTIVE MOVES	IMMOBILITY	SPINNING
C group	140.88 ±17.09	219.13 ± 17.09	19.38 ± 18.01
MD group	103.13 ± 17.39	256.88 ± 17.39	0

**Fig. 1:**
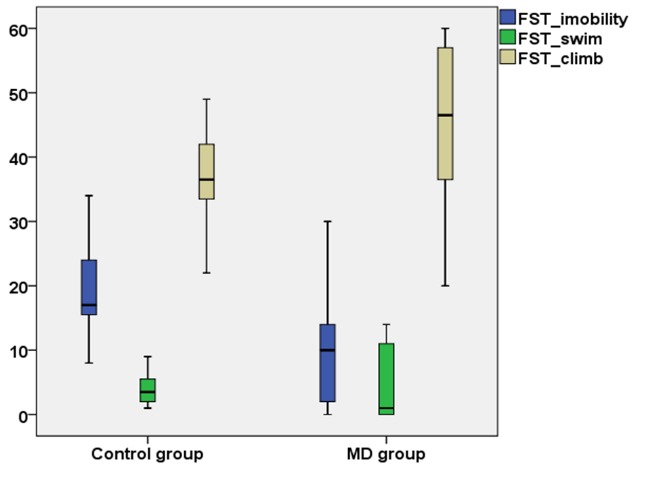
Variation of the behavioral parameters of Forced Swim Test in the two groups of rats. FST_Immobility - frequency of immobility in Forced Swim Test; FST_swim - frequency of swimming in Forced Swim Test; FST_climb - frequency of climbing in Forced Swim Test; MD group - melatonin deprived group

The variables measured in the FST were the *frequency of mobility, immobility* and *climbing*. The melatonin deprived group (MD) showed a higher frequency of immobility (p=0.05) compared to control group (C), while climbing and swimming behavior showed no statistical difference between groups (**Fig. 1**). 

**Fig. 2:**
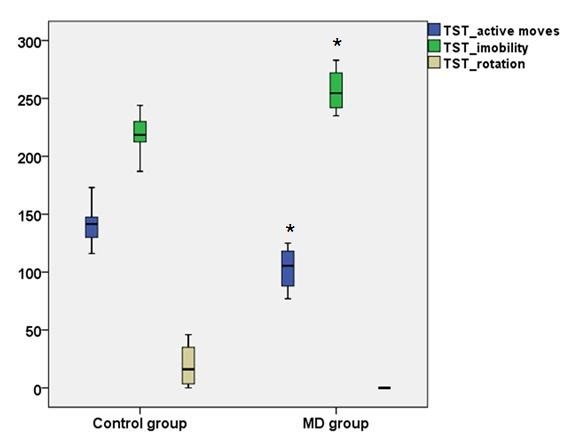
Variation of the behavioral parameters of Tail Suspension Test in the two groups of rats. TST_active moves - the mean time during any type of active moves in Tail Suspension Test; TST_immobility - the mean duration of immobility in Tail Suspension Test; TST_rotation - the mean time during spinning behavior in Tail Suspension Test; MD group - melatonin deprived group; * represents a p<0.05 in MD group vs. Control group

The variables measured in the TST were the *mean time during which rats performed any type of active moves (s), the mean duration of immobility, presence, absence and frequency of spinning behavior*. MD group was significantly less active than C group, having a lower mean duration of active moving (p=0.01) and a higher mean duration of immobility (p=0.001). The spinning movement was exclusively present in the C group and had a high frequency within this group (6 out of 8 rats) (**Fig. 2**).

## Discussion and conclusion

Melatonin secretion regulation is achieved by light/ dark exposure, with darkness stimulating melatonin secretion. Continuous light exposure alters nocturnal melatonin blood levels but it does not have any effect on diurnal melatonin concentration [**18**]. We have chosen E12-E21 gestational period for the functional pinealectomy model obtained by continuous light exposure of lab animals [**15**-**17**,**19**-**21**], as it was shown that melatonin concentration in dams raises progressively beginning with E12 until birth and goes down to nonpregnant levels immediately postpartum. Diurnal concentration is constant during pregnancy and it is not different from nonpregnant rats [**11**]. Fetuses are dependent on maternal melatonin production, as the functional maturity of the pineal gland is achieved postnatally. Melatonin levels in the fetal plasma are closely parallel to those in the maternal plasma and the rapid transfer of the maternal melatonin across the placenta has been demonstrated in several species, including rats [**19**-**29**].

Theoretically, both two screening behavioral tests currently used for assessing antidepressant medication efficacy, FST and TST, have the same clinical relevance, being conceptually related. In our study, FST behavior in the melatonin-deprived group turned out to be quite similar to controls, while TST showed significant differences between the two tested groups. Rats resulted from melatonin deprived pregnancies were less mobile and they lacked a special rotational movement (spinning) that control rats performed with a high frequency.

Induction of immobility by repeated ketamine treatment was reported to be present in the FST but not in the TST in mice [**33**]. The study concluded that both these models might follow different pathophysiological mechanisms and the excessive release of serotonin leads to overactivity of the 5HT2A system, which leads to enhanced immobility in the FST model, but induces hyperactivity in the TST model. Further, blockade of dopamine receptors by haloperidol aggravates the symptoms in both FST and TST. Clozapine pretreatment attenuates ketamine-induced immobility in the FST model, but shows conflicting results in the TST system. This pharmacological evaluation indicates that dopamine functioning is a necessity for the performance of mice in the FST, whereas both serotonergic and dopaminergic systems are involved in TST model [**33**].

Our study showed that melatonin deprivation during pregnancy leads to depressive like behavior in adult male offspring when animals are tested with TST, but with a normal appearance of the FST. This suggested that melatonin deprivation during pregnancy might cause a deficient serotonin signaling in the adult brain. 

Moreover, the present study showed differences of active movement in the TST between control rats, which performed (6 out of 8) a special spinning movement, and melatonin deprived rats, which showed no spinning. We did not find data in literature to explain the significance of this extra spinning movement, that was not previously described related to this test protocol, and to support its connection with melatonin and/ or serotonin signaling.

There were cases in which the active movements performed by the rats were recorded. These are 1) swinging - keeping its body straight, the rat continuously moves its paws in a vertical position and/ or moves its body from side to side, and 2) curling - the rodent engages in active twisting movements. As it is quite hard and not accurate to assess these movements without an automatic system, we have not taken them into consideration. Studies performed in mice differentiate between the two active behaviors, swinging and curling, and relate their variations to the use of different antidepressants and opioid drugs. While antidepressants have been shown to increase swinging behavior, they had no effect on curling, whereas opioids increased curling behavior. Importantly, both antidepressants and opioids diminish the immobility of the mice, the traditional measure of antidepressant-like activity in the TST [**34**].

In conclusion, the significant differences in TST, but not in FST, obtained in our study between the melatonin deprived and control groups, suggested that intrauterine melatonin deprivation might be linked to depressive-like behavior in the offspring. 

**Sources of funding**

Founding through POSDRU/107/1.5/S/82839.

**Disclosures**

None.
